# Radiation-Induced Angiosarcoma Arising in Bilateral Breast: A Case Report

**DOI:** 10.7759/cureus.68782

**Published:** 2024-09-06

**Authors:** Wakako Tsuji, Katsuhiro Yoshikawa, Fumie Fujisawa, Chikako Yamauchi, Akihiko Sugimoto

**Affiliations:** 1 Department of Breast Surgery, Shiga General Hospital, Moriyama, JPN; 2 Department of Plastic Surgery, Shiga General Hospital, Moriyama, JPN; 3 Department of Oncology, Shiga General Hospital, Moriyama, JPN; 4 Department of Radiation Oncology, Shiga General Hospital, Moriyama, JPN; 5 Department of Diagnostic Pathology, Shiga General Hospital, Moriyama, JPN

**Keywords:** bilateral radiation-induced angiosarcoma, breast-conserving therapy, recurrence, surgical resection with an adequate margin, taxane, wide skin grafting

## Abstract

Radiation-induced angiosarcoma is a highly aggressive malignancy. We encountered a case of angiosarcoma arising in the bilateral breast seven years after partial mastectomy and the last radiation therapy. As recommended, wide resection with skin grafting was performed to ensure negative surgical margins. After surgery, taxane-based chemotherapy was administered. However, adjuvant chemotherapy was discontinued because of drug-induced institutional pneumonia. Eighteen months postoperatively, angiosarcoma recurred in subcutaneous, lymph nodes, lungs, and bones. The disease progressed too rapidly, and the patient died two months after recurrence.

## Introduction

Causes of secondary angiosarcoma of the breast include chronic lymphedema after mastectomy with axillary dissection and radiation therapy following breast-conserving surgery. Angiosarcoma of the breast after postoperative radiotherapy is an aggressive malignancy that develops in previously irradiated areas [1]. The incidence of radiation-induced angiosarcoma is reported to be approximately 0.1% among breast cancer patients who underwent breast-conserving surgery followed by radiation therapy [2]. The shift from radical mastectomy to breast-conserving surgery has been postulated to result in an increasing incidence of radiation-induced angiosarcomas in the breast. In a Dutch population-based study, the median latency period from breast cancer treatment to the development of radiation-induced angiosarcoma was eight years (range, three to 20 years) [3]. Cutaneous biopsy is essential for the diagnosis of radiation-induced angiosarcoma [4], and the recommended treatment is surgical resection with wide margins. Recurrence rates are high, and the benefits of adjuvant therapy are unclear [5]. Here, we report a case of bilateral radiation-induced angiosarcoma that was treated with extensive resection followed by skin grafting.

## Case presentation

An 83-year-old woman presented to our hospital in August 2022 with bilateral breast nodules and skin redness. She had been diagnosed with metachronous bilateral breast cancer and underwent a right partial mastectomy and axillary dissection in April 2010. The pathological diagnosis was invasive ductal carcinoma, histological grade 2, estrogen receptor (ER) negative, progesterone receptor (PgR) negative, and human epidermal receptor 2 (HER2) negative (score 1+). The Ki67-labeling index was 50%, and the tumor stage was pT2 (26 mm) N2 (4/11)M0. As adjuvant chemotherapy, epirubicin plus cyclophosphamide (EC) was administered. However, she developed febrile neutropenia on day 13 of the first cycle and refused to continue chemotherapy. Subsequently, she received tegafur-uracil (UFT) for one year. In addition, radiation therapy was administered to the right breast, delivering a total dose of 50 Gy in 25 fractions. 

In August 2015, during her annual screening mammography and ultrasonography, an irregular low echoic mass was detected in her left breast. A core needle biopsy confirmed invasive ductal carcinoma. Subsequently, in September 2015, a left lumpectomy was performed. The pathological diagnosis was invasive ductal carcinoma, nuclear grade 1, ER+ (J score 3b), PgR+ (J score 3a), HER2- (score 1+), with a Ki67-labeling index of less than 5%. The tumor staging was pT1b (7 mm) N0 (0/1)M0. Radiation therapy was administered to the remnant left breast in a dose of 50 Gy over 25 fractions. She underwent adjuvant endocrine therapy with letrozole from October 2015 to October 2020, and annual screening was continued. 

**Figure 1:**
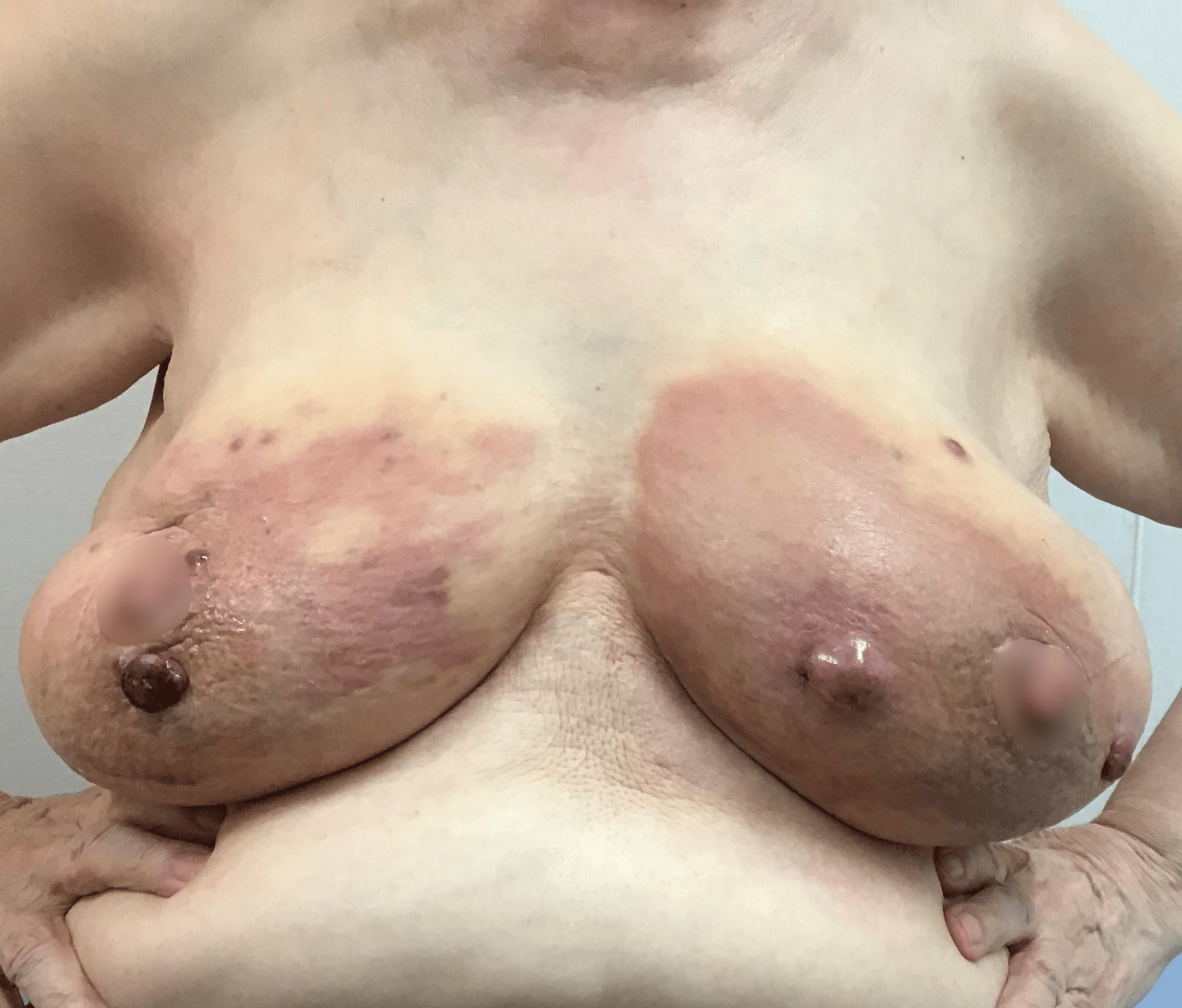
Clinical photograph. Irregular skin redness and multiple nodules are observed on both breasts.

In June 2022, she noticed an ecchymotic appearance on the left breast and skin redness that spread rapidly on both breasts (Figure 1).

**Figure 2:**
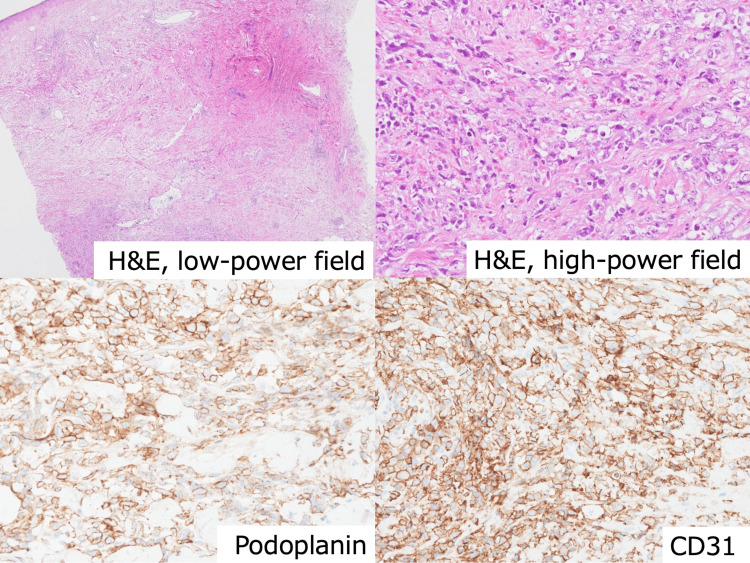
Microscopic pathological findings. Microscopic pathological findings from a skin biopsy (low- and high-power field of hematoxylin and eosin (H&E) staining, podoplanin, CD31). H&E staining shows vascular spaces lined by atypical cells with markedly pleomorphic vesicular nuclei and moderate eosinophilic cytoplasm in the deep derma. Immunohistochemical analyses show strong and diffuse membrane positivity for CD31 and podoplanin.

She visited the outpatient clinic in August 2022. Local recurrence was suspected, and a skin biopsy was performed. The pathological diagnosis was angiosarcoma, CD31+, CD34+, podoplanin+, Ki67-labeling index of 90%, pan-cytokeratin-, CK7-, CK5/6-, p63-, GATA3-, GCDFP-, ER-, PgR-, and HER2- (Figure 2).

**Figure 3:**
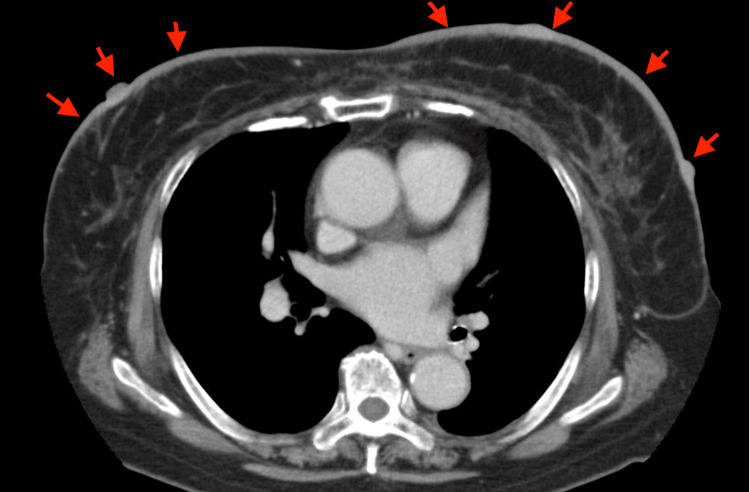
Chest CT imaging. Chest CT imaging shows bilateral breast skin thickness and multiple nodules (arrows). No distant metastasis is observed.

Computed tomography (CT) imaging showed no sign of distant metastasis or lymph node metastasis (Figure 3).

**Figure 4:**
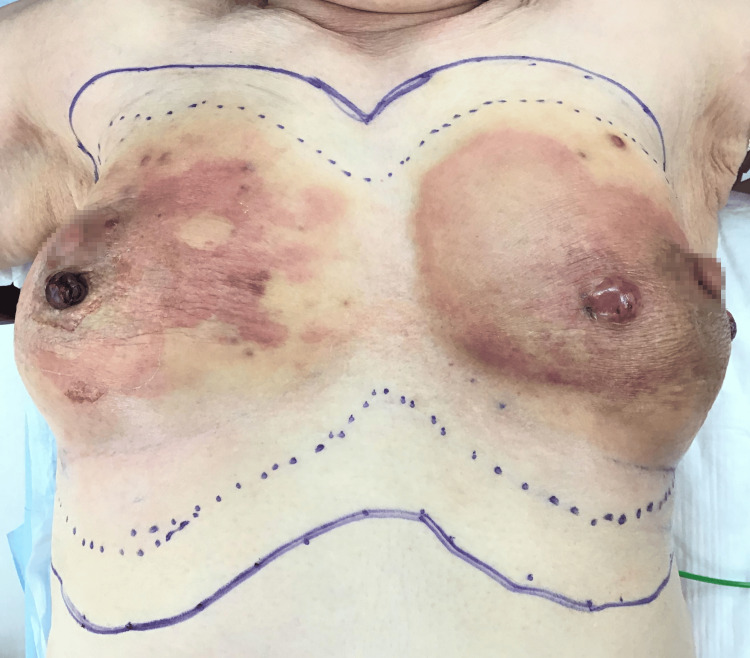
Skin incision design before surgery. Skin incision was drawn at least 3 cm margin from the skin disease.

**Figure 5:**
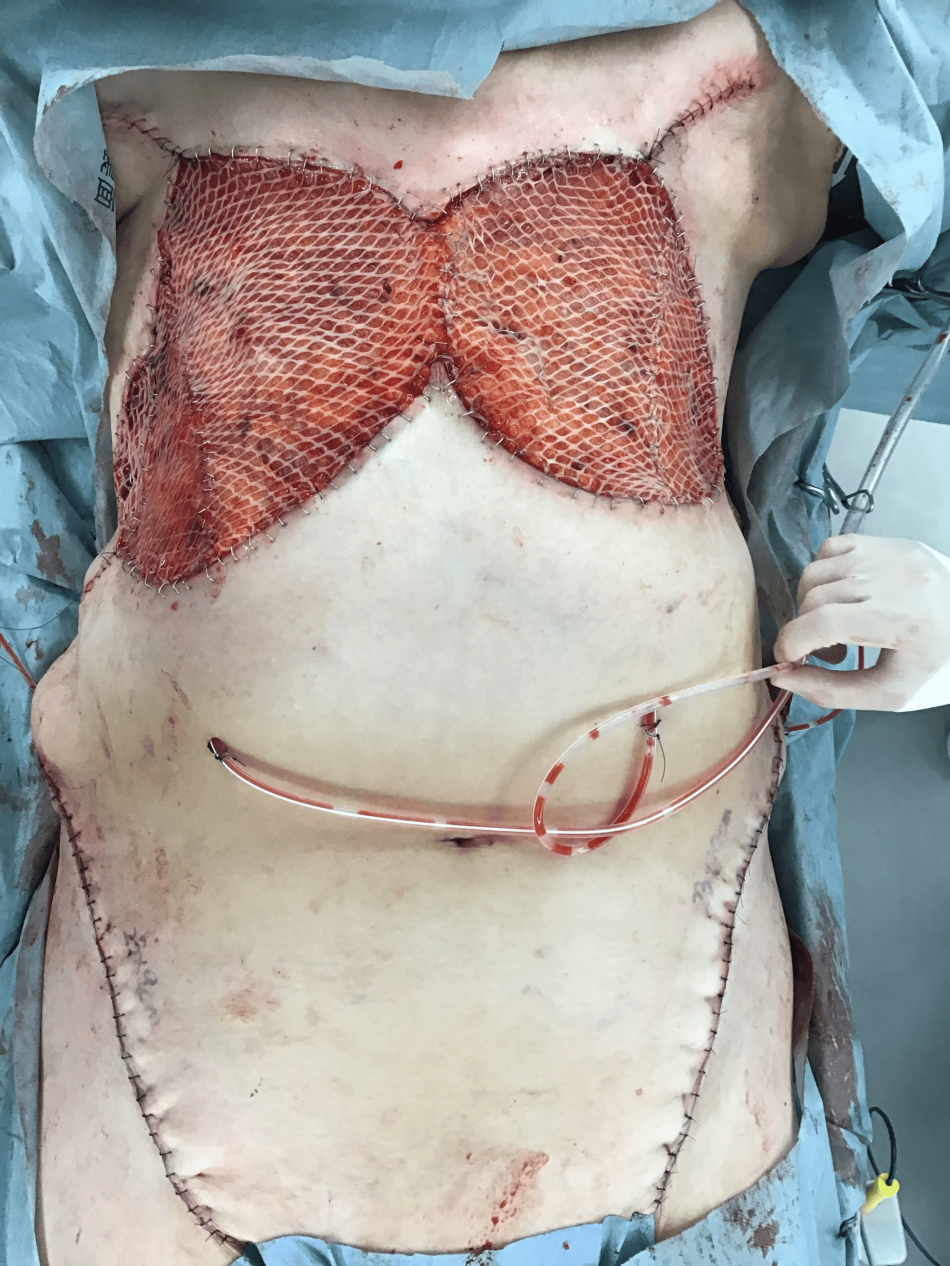
Appearance after resection and skin grafting. Bilateral total mastectomies were performed with a margin of 3 cm, and a skin graft was transplanted from the bilateral inguinal region.

In August 2022, bilateral total mastectomies were performed with a margin of 3 cm (Figure 4), and a skin graft was transplanted from the bilateral inguinal region (Figure 5).

**Figure 6:**
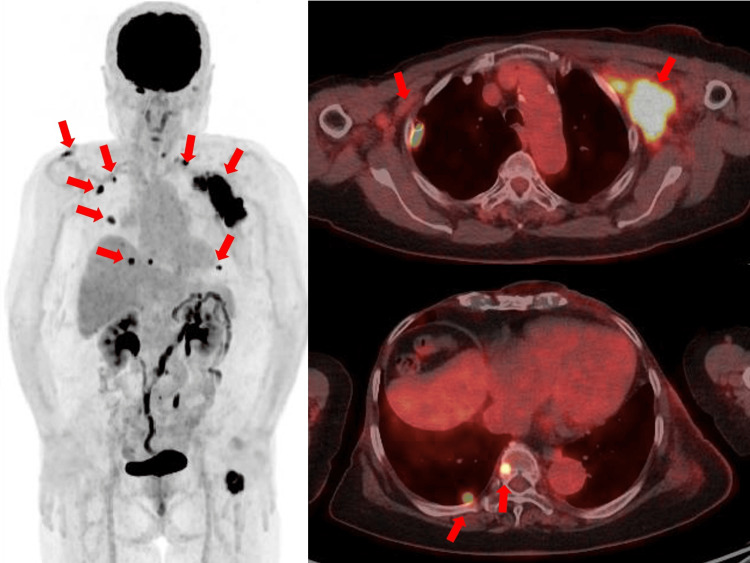
PET-CT imaging. PET-CT imaging shows recurrence in the left axilla, multiple lymph nodes, lung, and bone (arrows).

The pathological diagnosis was angiosarcoma with a negative margin for the peripheral and deep side. Weekly paclitaxel was administered as adjuvant chemotherapy. However, on day 8 of the first cycle, she discontinued the treatment because of alcohol intolerance. In October 2022, 85% of nab-paclitaxel was administered. On day 15 of the first cycle, she visited the outpatient clinic because of general fatigue. A chest CT revealed interstitial pneumonia. Subsequently, further continuous chemotherapy was discontinued, and we carefully monitored the patient. She noticed mild pain in the left axilla from January 2024: the axilla was swollen when the patient visited the outpatient clinic in February 2024. Chest CT imaging suggested local recurrence. A core needle biopsy of the left axilla was performed. Immunohistochemical examination showed CD31+, CD34+, ER-, and GATA3-. The pathological diagnosis was a recurrent post-radiation angiosarcoma. Positron emission tomography revealed a local recurrence in the left axilla and multiple metastases to the lymph nodes, lungs, and bones (Figure 6).

Radiotherapy was administered to the left axilla (39 Gy/13 fr) from February to March 2024. Daily oral administration of 600 mg of pazopanib was initiated on April 9. However, she stopped taking pazopanib four days after administration because of shortness of breath, fatigue, and melena. Chest CT showed a right pneumothorax and bilateral bloody pleural effusion, probably due to collapsed lung tumors. On April 22, shortness of breath worsened, and follow-up chest CT revealed bilateral pneumothorax, increased bilateral pleural effusion, and increased size of multiple hollowing lung metastases. The metastases spread rapidly, and we considered supportive care preferable for this patient. The patient died on April 25.

## Discussion

Surgical resection with an adequate wide margin is a basic treatment for radiation-induced angiosarcoma [6], and mastectomy with negative margins is considered the standard procedure. In this case, a 3 cm margin was designed to address the disease. Notably, no recommendation for the length of the safety margin is available. Mapping biopsy was considered to restore negative surgical margins [6]; however, immediate surgery was a priority for this patient. After wide resection, mesh skin grafting was applied from the inguinal region, as it was deemed necessary for this case. 

In the breast, both primary and secondary angiosarcomas exist. These entities appear similar under the microscope but differ in patient demographics and clinical presentation [7]. Primary angiosarcoma typically arises de novo and presents in younger patients within the breast parenchyma as an enlarging painless mass. On the other hand, secondary angiosarcomas in the breast develop as a consequence of prior radiation treatment. Radiation-induced angiosarcomas are secondary angiosarcoma, presenting as a rash or enlarging vascular lesion on the skin of the breast in older women [8].

Although radiation-induced angiosarcoma is described in the literature, the development of bilateral angiosarcoma is extremely rare. We could only identify one report from Sri Lanka [9]. This case was also diagnosed with synchronous bilateral breast carcinoma five years before the diagnosis of angiosarcoma. Our case suffered from metachronous bilateral breast cancers. Hereditary breast and ovarian cancer syndrome was suspected. In addition, we have to pay attention that radiation therapy is contraindication if the patient has TP53 pathogenic variants. Our case was 70 and 75 years old when she suffered from her first and second breast cancer, respectively. Li-Fraumeni syndrome was considered to be unlikely. In Japan, BReast CAncer gene (BRCA) testing is covered, but gene panel testing is not covered by the universal health insurance system. We recommended our patient genetic counseling followed by genetic testing. However, she did not want to take genetic counseling. It was unclear if she had genetic BRCA pathogenic variants.

There is no consensus on adjuvant chemotherapy; however, taxanes have been reported to be effective for advanced or unresectable angiosarcomas. Pazopanib, a vascular endothelial growth factor (VEGF) receptor tyrosine kinase inhibitor, has a reported response rate of 26.7% [10]. Taxanes had already been administered, and her Eastern Cooperative Oncology Group Performance Status score was 2 when the recurrence was pathologically diagnosed. Therefore, orally administered pazopanib was selected. However, disease progression was rapid. She stopped taking pazopanib on day 4 due to severe fatigue. CT imaging showed a right pneumothorax on April 15, 2024, which may have occurred as an adverse event of pazopanib. However, seven days later, bilateral pneumothorax was observed on CT. We believe that the disease progressed aggressively.

## Conclusions

We report a rare case of radiation-induced angiosarcoma arising in the bilateral breast after breast-conserving therapy for metachronous breast cancer. Long-term disease control could not be achieved despite complete resection with a negative margin, followed by wide skin grafting and subsequent chemotherapy. Furthermore, post-recurrence survival was quite short, although radiotherapy and chemotherapy were given. Radiation-induced angiosarcomas are highly aggressive.
